# METTL3-mediated m^6^A modification promotes ferroptosis in adenomyosis through GPX4 in a YTHDF1-dependent manner

**DOI:** 10.1530/REP-25-0251

**Published:** 2025-09-19

**Authors:** Ying Tang, Zili Li, Xin Zhao, Xiaomin Niu, Shuang Ning, Liya Liu, Zheying Liu, Jingru Zhang, Qian Zhao, Liping Han

**Affiliations:** ^1^ Department of Gynecology and Obstetrics, The First Affiliated Hospital of Zhengzhou University, Zhengzhou, China; ^2^ Genetic and Prenatal Diagnosis Center, Department of Gynecology and Obstetrics, The First Affiliated Hospital of Zhengzhou University, Zhengzhou, China

**Keywords:** Adenomyosis, Ferroptosis, m^6^A modification, METTL3, GPX4

## Abstract

Adenomyosis, a prevalent gynecologic disorder affecting women of reproductive age, is characterized by the presence of ectopic endometrial tissue within the myometrium. The involvement and underlying mechanisms of ferroptosis in adenomyosis have not been fully elucidated. Recently, m^6^A RNA modification has been found to regulate various biological processes. This study aimed to investigate the status of ferroptosis in adenomyosis and explore how m^6^A modification regulates key genes associated with ferroptosis. The research revealed ferroptosis present in both eutopic and ectopic endometrial tissues of individuals with adenomyosis. In addition, a decrease in RNA m^6^A modification levels and a reduction in GPX4 protein expression were observed. GPX4 may serve as a biomarker reflecting the severity of adenomyosis, as it showed a significant negative correlation with CA125 levels, uterine size, and the severity of dysmenorrhea in patients. Mechanistically, the study demonstrated that the downregulation of METTL3 in endometrial stromal cells results in reduced m^6^A modification on *GPX4* mRNA. Consequently, *GPX4* mRNA translation is downregulated by YTHDF1, leading to ferroptosis in eutopic and ectopic endometrial cells. These findings contribute to a deeper understanding of the mechanisms underlying adenomyosis and provide valuable insights into potential therapeutic strategies targeting ferroptosis in the management of adenomyosis.

## Introduction

Adenomyosis (AM), a common gynecologic disease characterized by the presence of endometrial glands and stroma within the myometrium, can significantly reduce quality of life (Kho *et al.* 2021). Women with adenomyosis suffer from abnormal uterine bleeding (AUB) because of enlarged uterus, chronic pelvic pain, dysmenorrhea, dyspareunia, and infertility (Peric & Fraser 2006). The prevalence of adenomyosis is unknown. It was reported that adenomyosis affects 20–35% reproductive-aged women and 8.8–61.5% patients undergoing hysterectomy (Upson & Missmer 2020). Despite advancements in imaging techniques, the true prevalence may still be underestimated. Several theories, including the tissue injury and repair (TIAR) theory, *de novo* metaplasia theory, and ‘from outside to inside invasion’ theory, have been proposed to elucidate the mechanisms underlying adenomyosis (Zhai *et al.* 2020*b*). But these theories cannot fully explain the pathogenesis of adenomyosis, and adenomyosis is still wrapped in mystery (Munro 2021).

Ferroptosis, a form of cell death driven by iron-dependent lipid peroxidation, was first coined by Stockwell in 2012 and is now proved to participate in various biological processes (Stockwell 2022). Its occurrence is modulated by intracellular iron overload. The increase of intracellular free divalent iron (Fe^2+^) generates reactive oxygen species (ROS) through the Fenton reaction, leading to lipid peroxidation and membrane damage (Dixon & Olzmann 2024). While endometriosis and adenomyosis are interconnected diseases, it is yet premature to definitively assert that they represent distinct phenotypes of the same disease process (Habiba *et al.* 2023). Research on ferroptosis in endometriosis has also yielded conflicting results. On one hand, ferroptosis is activated by an iron-rich environment in ectopic lesions (Ng *et al.* 2020), which could aggravate inflammation and promote disease progression (Li *et al.* 2022, Ni *et al.* 2022, Zhang *et al.* 2022). On the other hand, ectopic endometrial tissues resist ferroptosis through multiple mechanisms (Dong *et al.* 2023, Wu *et al.* 2023, Chen *et al.* 2024). However, unlike ovarian chocolate cyst and abdominal endometriosis, there is no existing evidence about iron overload environment, oxidative stress, and ferroptosis in adenomyosis and whether ferroptosis occurs in adenomyosis such as endometriosis requires further investigation.

m^6^A RNA modification is the most abundant modification, which is defined as the methylation of adenosine at the 6-position in mRNA and some noncoding RNA (Deng *et al.* 2018). The process of RNA methylation is controlled by methyltransferases (writers), demethylases (erasers), and recognition factors (readers) (Zaccara *et al.* 2019). In recent years, there has been growing interest in exploring the role of m^6^A modification in the pathophysiological mechanisms of female reproductive system disorders (Chen *et al.* 2022, Zhang & Liu 2022, Huang & Chen 2023). But only one study utilizing bioinformatics methods indicated that individuals with adenomyosis exhibit significant reductions in m^6^A methylation levels (Zhai *et al.* 2020*a*). Further research is needed to fully elucidate the precise underlying mechanisms involved in m^6^A modification in the context of adenomyosis.

Numerous studies have indicated that m^6^A modification is involved in the regulation of ferroptosis. It has been demonstrated across various tumor studies that ferroptosis-related proteins such as SLC7A11 (Qiao *et al.* 2024), SLC3A2 (Ma *et al.* 2021), and TFRC (Liu *et al.* 2023) contain m^6^A modification sites on their mRNAs and are regulated by m^6^A. Therefore, we speculate that similar regulatory mechanisms may exist in adenomyosis. Here, we aimed to clarify the phenomenon of ferroptosis, investigate the relationship between m^6^A modification and ferroptosis in adenomyosis, and explore its regulatory mechanisms to lay the foundation for therapy of adenomyosis based on ferroptosis.

## Materials and methods

### Study design

This cohort study was conducted at the Department of Gynecology in The First Affiliated Hospital of Zhengzhou University from December 2023 to September 2024. Ethical approval was obtained (No. 2024-KY-0731-001). All of the subjects included in our study signed an informed consent form before research recruitment.

The adenomyosis group included women 30–55 years of age whose preoperative diagnoses were established using transvaginal ultrasound or magnetic resonance imaging; and with postoperative pathological confirmation of adenomyosis after surgical treatment. Patients with malignancy or atypical endometrial hyperplasia, or those with a history of hormonal therapy or intrauterine device placement within 6 months before surgery, were excluded. Age-matched women diagnosed with benign gynecological conditions, such as hydrosalpinx, infertility, or uterine fibroids, served as controls. All surgeries for both patients and controls were performed using minimally invasive techniques (MIS).

Samples in the adenomyosis group were collected from both the endometrium and myometrium, while control samples included only endometrial tissue. Baseline characteristics of patients in terms of age, body mass index (BMI), gravidity, abortions, anemia, dysmenorrhea severity, CA125 levels, CA199 levels, and uterine size were also collected.

### RNA-seq

Total RNA was isolated using Trizol reagent. Then mRNA sample library construction and high-throughput sequencing were performed by Hangzhou KaiTai Biotechnology Co., Ltd. In brief, polyadenylated RNA was enriched from total RNA. mRNA samples were fragmented using RNA Fragmentation Reagents (AM8740; Ambion, USA) and used for library construction and performed high-throughput sequencing on Illumina NovaSeq 6000.

### Transmission electron microscopy

The fresh endometrium and myometrium tissue from patients with adenomyosis were cut into small pieces, and 2.5% glutaraldehyde was added to fix for 5 min. After centrifugation at 700 *g* for 3 min, the supernatant was discarded. We prepared 1% osmic acid with 0.1 M phosphate buffer (PB) at pH 7.4, which was fixed at room temperature for 2 h, rinsed with 0.1 M PB, dehydrated at room temperature with 30–100% alcohol and 100% acetone, and embedded. After that, the plate was polymerized in an oven at 60°C, and the resin block was cut into 60–80 nm ultra-thin slices.

### Measurement of malondialdehyde (MDA)

The concentration of MDA, one of the end products of lipid peroxidation, was detected with the Malondialdehyde (MDA) Content Assay Kit, Micromethod (D799762, Sangon Biotech, China) following the manufacturer’s instructions. MDA levels were measured at an absorbance of 532 nm by using a multimode microplate reader (BioTek Instruments, USA).

### Measurement of iron content in tissues

Fresh tissues were rinsed multiple times with PBS until no obvious blood residue remained, then weighed to ensure each sample had a net weight of 100 mg. The concentration of total iron and ferrous ion was detected with the Iron Assay Kit, Colorimetric (I291, Dojindo, Japan). Standards, test samples, and blank control solutions were prepared according to the kit instructions. Absorbance was measured at 596 nm using a microplate reader (BioTek Instruments, USA).

### Western blotting

Protein samples were prepared by using RIPA buffer (Solarbio, China), and SDS-PAGE gels were used. PVDF membranes (Millipore, USA) were used for transfer and blocked with 5% skim milk for 2 h at 25°C. The corresponding primary antibodies against GAPDH (10494-1-AP, Proteintech, China), METTL3 (15073-1-AP, Proteintech, China), and GPX4 (67763-1-AP, Proteintech, China) were incubated at 4°C overnight, and then the secondary antibody was incubated at 25°C for 1 h. The relative expression level of protein was calculated using a chemiluminescence imaging system. The uncropped western blot images are shown in Supplementary Fig. S3 (see section on Supplementary materials given at the end of the article).

### Immunohistochemical (IHC) staining

Endometrium and myometrium specimens from patients were detected by IHC. Uterus from mice were detected by immunofluorescence (IF). In short, 1% hydrogen peroxide was used as a blocker to block antigen retrieval. The corresponding primary antibody was incubated at 4°C overnight, followed by incubating the secondary antibody at room temperature for 30 min, then using DAB chromogenic agent. For IF, the sections were then incubated with the FITC- or Cy3-conjugated secondary antibodies (1:500–1,000) for 1 h at RT, washed with PBS three times, and counterstained with DAPI (1:1,000) for 10 min. Stained sections were scanned using the 3DHISTECH imaging system (Hungary). Each section was measured in three different fields. The mean optical density was the mean of the integrated optical density of three nonoverlapping fields.

### m^6^A dot blot assay

Total RNA was extracted with Trizol reagent and denatured by heating at 100°C for 10 min, followed by chilling on ice immediately. Next, RNA was spotted on a Nylon Transfer Membrane (TM-NY-S-45, Labselect, China) and cross-linked by 365 nm UVP for 30 min. m^6^A levels were measured using an anti-m^6^A antibody (68055-1-Ig, Proteintech, China). Nylon membrane blocking and antibody incubation refer to western blotting. The results were also detected as in western blotting. After exposure, the membrane was transferred to 0.02% methylene blue dye buffer and then pictures were taken in the bright field.

### Cell culture

Primary eutopic endometrial stromal cells (EuESCs) were isolated from the endometrium of women with adenomyosis. Briefly, the tissues were washed with PBS, finely diced, and enzymatically digested using collagenase IV for 1 h at 37°C. Epithelial cells were separated through a 40 μm sieve, and stromal cells were subsequently enriched. EuESCs were cultured in DMEM/F12 (Gibco, USA) supplemented with 10% fetal bovine serum (FBS) and 1% penicillin/streptomycin within a humidified 5% CO^2^ environment at 37°C. The purity of endometrial stromal cells was confirmed by immunofluorescent staining using vimentin (GB121308, Servicebio, China) and cytokeratin 1 (GB111017, Servicebio, China), respectively, as markers of stromal and epithelial cells. The number of vimentin-positive stained cells was above 95% in our study (Fig. S1B).

### RNA interference (RNAi) and overexpression

METTL3 siRNA and control siRNA were purchased from GenePharma (China). METTL3-overexpressing plasmids (pcDNA3.1 vector) were generated by GenePharma (China). Full-length GPX4 with 5′UTR and 3′UTR overexpressing plasmids (pcDNA3.1 vector) was constructed by Youbio (Shenzhen, Guangzhou, China). The mut GPX4 plasmids were constructed using the Mut Express II Fast Mutagenesis Kit V2 from Vazyme (Nanjing, Jiangsu, China). All the plasmids were verified by Sanger sequencing (Sangon Biotech, China). The sequences of plasmids and siRNA are listed in Supplementary Table S3.

Before transfection EuESCs in the logarithmic growth phase were inoculated into culture dishes, and cell fusion reached 60–80% confluence on the second day. Lipofectamine 3000 (Invitrogen, USA) was applied to prepare the mixture of METTL3-siRNA, control-siRNA, pcDNA-METTL3, or pcDNA-GPX4-Lipofectamine™3000 as the instructions. The cells were collected 48 h after transfection.

### Flow cytometry and measurement of ROS

Intracellular ROS levels were detected with a fluorescent probe DCFH-DA (S0033S, Beiotime, China) as the manufacturer’s instructions. In brief, EuESCs were seeded into 6-well plates and transfected with METTL3 siRNA or pcDNA3.1-METTL3 for 48 h. Then they were exposed to erastin 10 μM (HY-15763, MCE, China) or ferrostatin-1 1 μM for 24 h (HY-100579, MCE, China). Finally, cells were incubated with DCFH-DA at a final concentration of 10 μM in medium without FBS at 37°C for 0.5 h and washed three times using medium. ROS levels were assessed using a flow cytometer (CytoFLEX, Beckman Coulter, USA).

### MeRIP-seq and MeRIP-qPCR

Total RNA isolated from endometrium and myometrium tissue in adenomyosis patients was used for MeRIP-seq. EuESCs infected with METTL3-siRNA and control-siRNA were harvested at 48 h post-infection for MeRIP-qPCR. The mRNA was further purified using the Seq-StarTM poly(A) mRNA Isolation Kit (AS-MB-006-01, Arraystar, USA). After fragmentation with RNA fragmentation reagent, the anti-m^6^A antibody (Synaptic Systems, 202003, Germany) was used for immunoprecipitation. Both input and immunoprecipitation RNA samples were then subjected to the sequencing library preparation using the KAPA Stranded mRNA-seq Kit (Roche, Switzerland). Clustered libraries were loaded onto reagent cartridges and forwarded to sequencing run on Illumina NovaSeq 6000 system or MeRIP-qPCR analysis. Supplementary Table S4 lists the primers used in this assay.

### RIP-qPCR

EuESCs were seeded in two 10 cm dishes at a density of 1 × 10^6^ cells/mL. Then cells were infected with METTL3-siRNA, control-siRNA, or mut GPX4 plasmids for 48 h. Cells were lysed using the RIP-Assay Kit (Bes5101, Bersinbio, China). The anti-YTHDF1 antibody (17479-1-AP, Proteintech, China) or IgG was used for immunoprecipitation. After that, the cells were treated with protein A/G beads. The RNA from input and immunoprecipitated samples was isolated with the TRIzol reagent and then analyzed using qRT-PCR. Supplementary Table S4 lists the primers used in this assay.

### HE staining and Prussian blue staining

Endometrium and myometrium tissue from patients, as well as uterus from mice, were fixed with 4% paraformaldehyde for 24 h. After dehydration in alcohol and xylene, and paraffin embedding the tissues immediately, the tissues were cut into 5 μm slices. For HE staining, slides were stained with hematoxylin and eosin (H&E). For Prussian blue staining, slides were immersed for 1 h in 1% potassium ferricyanide. Stained sections were scanned using the 3DHISTECH imaging system (Hungary).

### Animal experiments

All CD-1 mice (8 weeks) were provided by Vital River Laboratory Animal Technology Co. Ltd (China) and maintained under specific pathogen-free conditions. All the mouse experiments were approved by the institutional ethics committee of The First Affiliated Hospital of Zhengzhou University as mentioned above.

For the adenomyosis group, 20 female neonatal mice were orally administered 1 mg/kg tamoxifen from day 2 to day 5 after birth. A control group of 20 female neonatal mice received an equivalent volume of solvent only. Once the mice reached 3 weeks of age, they were weaned and separated from their dams. Starting at 6 weeks of age, the female mice from both the adenomyosis and control groups were randomly allocated into four subgroups, with five mice in each subgroup. For each group, every mouse received erastin 40 mg/kg (HY-134836, MCE, China), ferrostatin-1 5 mg/kg (HY-100579, MCE, China), or STM2457 50 mg/kg (HY-134836, MCE, China) by intraperitoneal injection over a 2-week period. The control group was treated similarly (one mouse that received erastin died during injection) (Fig. 6A). All the mice at the eighth week were euthanized, and the uterus was harvested for the following experiment.

### Statistical analysis

In the analysis of clinical data, the *χ*^2^ test and nonparametric tests were utilized to compare the differences between two groups. Statistical analyses were performed using SPSS version 25. Experimental data were analyzed with GraphPad Prism 9.0 software and were duplicated no less than three times independently. A one-way ANOVA was used to compare differences between three groups. An unpaired two-tailed Student’s *t*-test was used to compare differences between two groups. For all the statistical analyses, *P* < 0.05 was considered statistically significant.

## Results

### Baseline characteristics of clinical specimens

In our study, a total of 75 patients were included. The adenomyosis group comprised 40 women who underwent combined hysteroscopic and laparoscopic surgery or laparoscopic hysterectomy. Control samples included endometrium tissue from three women diagnosed with cervical intraepithelial neoplasia (CIN)-III, ten women with uterine leiomyoma, and 22 women who underwent hysteroscopy due to AUB. Information about these patients and endometrial phase is shown in Supplementary Table S1. There were no significant differences observed in age, BMI, or anemia severity between the adenomyosis group and the control group. Patients in the adenomyosis group had a higher median gravidity and number of abortions compared to the control group. In addition, adenomyosis patients exhibited significantly more severe dysmenorrhea, elevated CA125 levels, and a notably larger uterine size, with statistically significant differences compared to the control group (Supplementary Table S2).

### Ferroptosis occurs in the endometrium and myometrium of patients with adenomyosis

**Figure 1:**
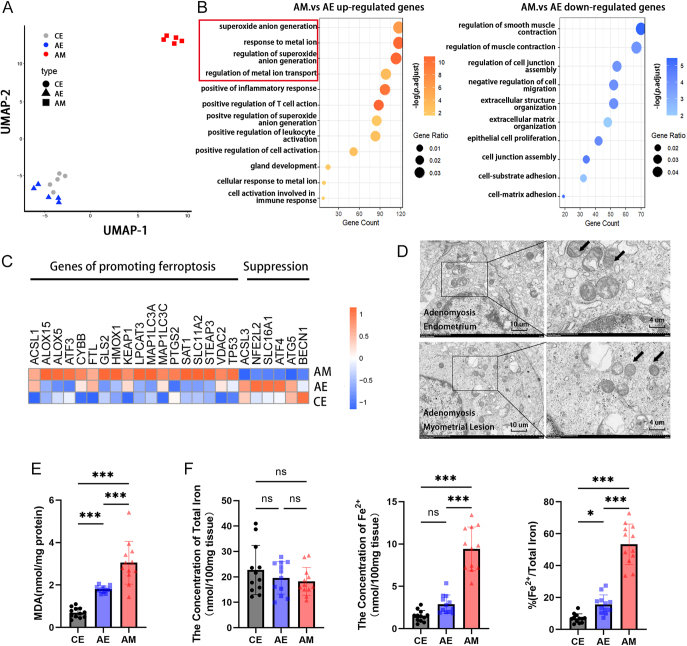
Ferroptosis occurrence in the endometrium and myometrium of patients with adenomyosis. (A) UMAP analysis of transcriptome sequencing results. (B) GO analysis of differentially expressed genes. (C) Heatmap of differentially expressed genes related to ferroptosis. (D) Transmission electron microscopy analysis of mitochondrial ultrastructure in the endometrium and myometrium of adenomyosis. Mitochondria structures are indicated by arrows. Scale bar = 4 and 10 μm. (E) Malondialdehyde (MDA) levels in specimens from three groups (*n* = 12). ***P < 0.001. (F) The concentration of total iron, ferrous iron content, and Fe^2+^ percentages in CE, AE, and AM (*n* = 12). *P < 0.05, ***P < 0.001. CE, control endometrium; AE, adenomyosis endometrium; AM, adenomyosis myometrial lesion.

To investigate the potential factors and mechanisms involved in iron levels and determine the presence of ferroptosis in adenomyosis, we utilized endometrial samples from control groups as well as endometrial and myometrial lesion samples from adenomyosis patients for RNA-sequencing (RNA-seq) analysis. Our findings revealed distinct RNA expression patterns in myometrial lesions compared to normal endometrium and adenomyosis endometrium (Fig. 1A). Subsequently, gene ontology (GO) analysis was conducted between the myometrial lesion group and the adenomyosis endometrial group, highlighting the enrichment of upregulated genes associated with ion metabolism and oxidative stress pathways (Fig. 1B). Next, the expression levels of ferroptosis-related genes were examined. Genes that promote ferroptosis, such as *ACSL1, ALOX15, STEAP3*, and *SLC11A2*, were upregulated in adenomyosis (Fig. 1C). Validation of ferroptosis occurrence in the endometrium and myometrium of patients was performed by assessing mitochondrial morphological structures via transmission electron microscopy. Mitochondria in myometrial lesions appeared shorter with increased membrane density (Fig. 1D). In addition, the level of malondialdehyde (MDA) was significantly higher in adenomyosis myometrial lesions compared to endometrium or control groups (Fig. 1E). To further investigate the presence of iron overload in adenomyosis, we conducted Prussian blue staining on three kinds of specimens, which revealed no significant blue-stained particles (Fig. S1A). However, we observed a notably higher content of ferrous iron in the myometrial lesion of adenomyosis (Fig. 1F). Overall, these findings indicate that adenomyosis exhibits characteristics of ferroptosis.

### GPX4 protein expression is decreased and correlated with advanced state in patients with adenomyosis

**Figure 2:**
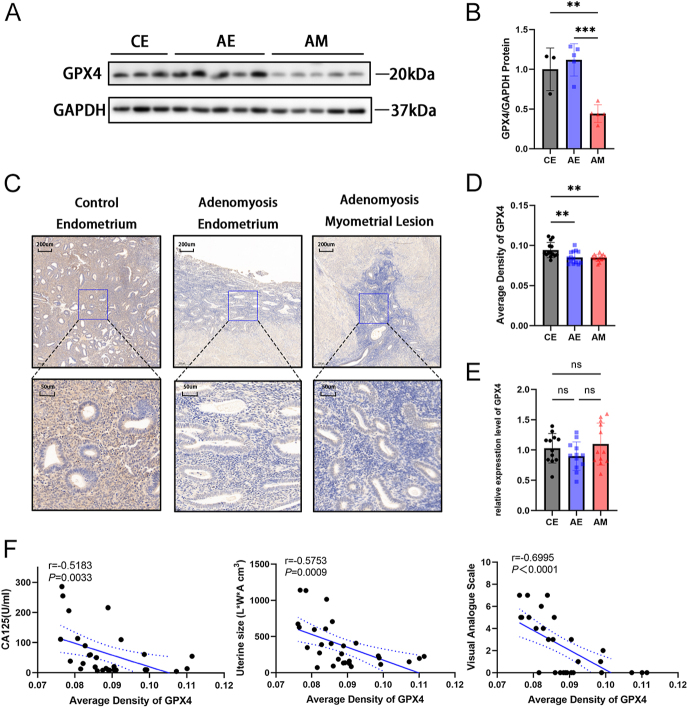
GPX4 protein expression is decreased in adenomyosis. (A) GPX4 protein levels determined by western blotting. (B) Protein levels normalized to GAPDH. **P < 0.01, ***P < 0.001. (C) Representative immunohistochemical staining for GPX4 in CE, AE, and AM. Scale bar = 200 and 50 μm. (D) Average optical density analysis of GPX4 immunohistochemistry staining (*n* = 15). **P < 0.01. (E) *GPX4* mRNA levels by RT-qPCR (*n* = 12). (F) Correlation between GPX4 expression and CA125 levels, uterine size, and dysmenorrhea severity in adenomyosis patients. GPX4 expression was detected by IHC (*n* = 30). CE, control endometrium; AE, adenomyosis endometrium; AM, adenomyosis myometrial lesion; L, length diameter; W, width diameter; A, anteroposterior diameter.

Glutathione peroxidase 4 (GPX4) is a key gene in the ferroptosis pathway, but our RNA-seq results showed no significant differences in *GPX4* mRNA expression. Therefore, we further explored the expression level of GPX4. At the protein level, western blot showed that GPX4 was lowly expressed in the myometrial lesions of adenomyosis (Fig. 2A and B). Similarly, immunohistochemistry showed the same results (Fig. 2C and D). An interesting phenomenon is that the mRNA level of GPX4 showed no significant difference when more specimens were used (Fig. 2E). Classic clinical manifestations of adenomyosis include uterine enlargement, secondary progressive dysmenorrhea, and elevated serum CA125. The visual analog scale is used to assess the severity of dysmenorrhea. A greater severity of the aforementioned clinical indicators corresponds to a more advanced disease state in patients with adenomyosis. We also explored the correlation between GPX4 expression and those clinical indicators. There was a significant negative correlation between GPX4 expression and CA125 levels, uterine size, and dysmenorrhea severity in adenomyosis (Fig. 2F).

### The m^6^A methylation level of adenomyosis is reduced because of the downregulation of METTL3

**Figure 3:**
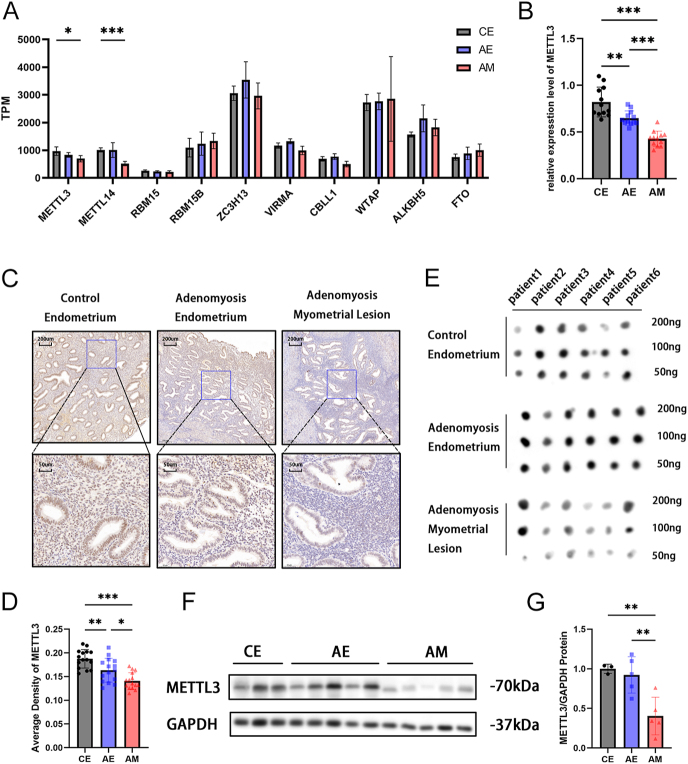
The m^6^A methylation level of adenomyosis is reduced because of the downregulation of METTL3. (A) Transcripts per million of m^6^A regulators between CE, AE, and AM. *P < 0.05. (B) The mRNA level of *METTL3* (*n* = 12). **P < 0.01, ***P < 0.001. (C) Representative immunohistochemical staining for METTL3 in three groups. Scale bar = 200 and 50 μm. (D) Average optical density analysis of METTL3 immunohistochemistry staining (*n* = 15). *P < 0.05, **P < 0.01, ***P < 0.001. (E) Dot blot assay using m^6^A antibody detected in three groups. (F) Protein levels of METTL3 in three groups by western blotting. (G) The protein levels normalized to GAPDH. **P < 0.01. CE, control endometrium; AE, adenomyosis endometrium; AM, adenomyosis myometrial lesion.

The above experiments reveal the presence of ferroptosis in the myometrial lesions of adenomyosis. We found that GPX4 protein expression is decreased while no significant differences were observed in GPX4 mRNA expression. To explore its regulatory mechanism, our focus turned to m^6^A RNA modification. Therefore, we conducted further analysis on the expression levels of m^6^A regulators, including ‘writers’, ‘erasers’, and ‘readers’, using RNA-seq. The results revealed that the methyltransferase-like protein 3 (METTL3) and methyltransferase-like protein 14 (METTL14) were downregulated (Fig. 3A). The methyltransferase complex catalyzed m^6^A modification by METTL3 and METTL14 and a regulatory subunit WTAP. METTL3, as a key component of the catalytic process, can directly affect the total methylation level of m^6^A. We mainly focused on METTL3 in the following experiments. This finding was confirmed through RT-PCR (Fig. 3B), IHC (Fig. 3C and D), and western blotting (Fig. 3F and G). These findings suggested that the expression of METTL3 was downregulated both at the mRNA and protein levels. Furthermore, a more pronounced decrease in METTL3 was identified in the myometrial lesions of adenomyosis. To validate the decrease in RNA methylation attributed to METTL3, dot blot analysis demonstrated that the total m^6^A methylation level was significantly lower in adenomyosis myometrial lesions compared to the other two groups (Fig. 3E).

### The downregulation of METTL3 promotes ferroptosis in EuESC cells

**Figure 4:**
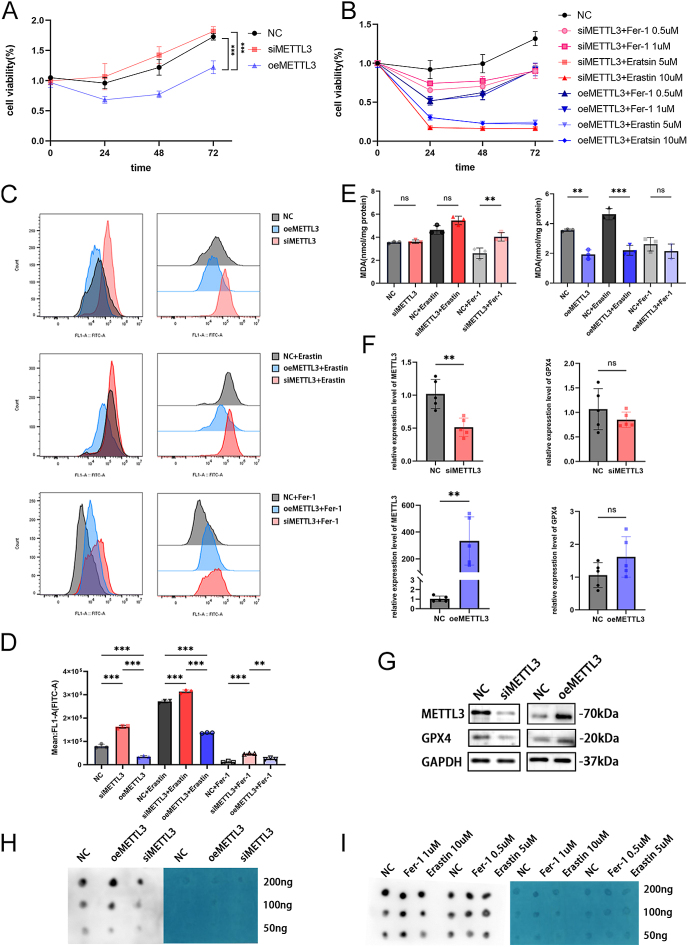
The downregulation of METTL3 promotes ferroptosis in EuESC cells. (A) Cell viability of EuESCs transfected with METTL3 siRNAs or METTL3-overexpressing plasmids for 24, 48, and 72 h. (B) Cell viability of EuESCs following METTL3 knockdown or overexpression and subsequent treatment with erastin or fer-1 for 24, 48, and 72 h. (C) Assessment of ROS levels in EuESCs after METTL3 knockdown or overexpression and treatment with erastin or fer-1 for 24 h using flow cytometry. (D) Statistical analyses of ROS from three independent experiments are shown. **P < 0.01, ***P < 0.001. (E) MDA levels of EuESCs got METTL3 knocked down or overexpressed and treated with erastin or fer-1 for 24 h (*n* = 3). **P < 0.01, ***P < 0.001. (F) The mRNA levels of *METTL3* and *GPX4* after getting METTL3 knocked down or overexpressed (*n* = 5). **P < 0.01. (G) Protein levels of METTL3 and GPX4 after getting METTL3 knocked down or overexpressed. The protein levels normalized to GAPDH are shown in Fig. S1C. (H) Dot blot assay using m^6^A antibody on cells with METTL3 knocked down or overexpressed. (I) Dot blot assay using m^6^A antibody after treating cells with erastin or fer-1 for 24 h.

To further investigate the relationship between ferroptosis and m^6^A methylation, first, we isolated primary eutopic endometrial stromal cells (EuESCs) then knocked down METTL3 or got METTL3 overexpressed. An interesting phenomenon we found is that cell viability was lower when overexpressing METTL3 than with si-METTL3 (Fig. 4A), whereas the cells with METTL3 knocked down were more susceptible to the ferroptosis inducer erastin than those overexpressing METTL3. At the same time, the ferroptosis inhibitor ferrostatin-1 could effectively rescue cell viability caused by erastin (Fig. 4B). Due to the lack of significant differences observed when varying concentrations of erastin or ferrostatin-1 were used, we opted to utilize 10 μM erastin and 1 μM ferrostatin-1 for subsequent experiments.

As anticipated, the results of the ROS assay showed a similar trend. ROS levels were higher in cells with si-METTL3 than in those with oeMETTL3, consistently observed regardless of treatment with erastin or ferrostatin-1 (Fig. 4C and D). Although there was no significant difference in MDA levels between the negative control (NC) and siMETTL3, treatment with ferrostatin-1 demonstrated that knocking down METTL3 led to higher MDA levels. This phenomenon was also confirmed in experiments involving the overexpression of METTL3 (Fig. 4E). Subsequent focus was directed toward GPX4. Although no significant difference was detected in GPX4 mRNA levels after METTL3 knockdown, western blotting indicated a reduction in GPX4 protein levels. Similar to the results of METTL3 knockdown, overexpressing METTL3 did not elicit changes in *GPX4* mRNA levels; GPX4 protein levels were increased (Fig. 4F and G). In addition, we identified that knocking down METTL3 could reduce the m^6^A methylation level of cells; however, promoting or inhibiting ferroptosis did not influence the overall level of m^6^A methylation (Fig. 4H and I). Overall, these results collectively suggest that knockdown of METTL3 could aggravate ferroptosis.

### The reduction of m^6^A-methylated *GPX4* mRNA led to decreased *GPX4* mRNA translation in a YTHDF1-dependent manner

**Figure 5:**
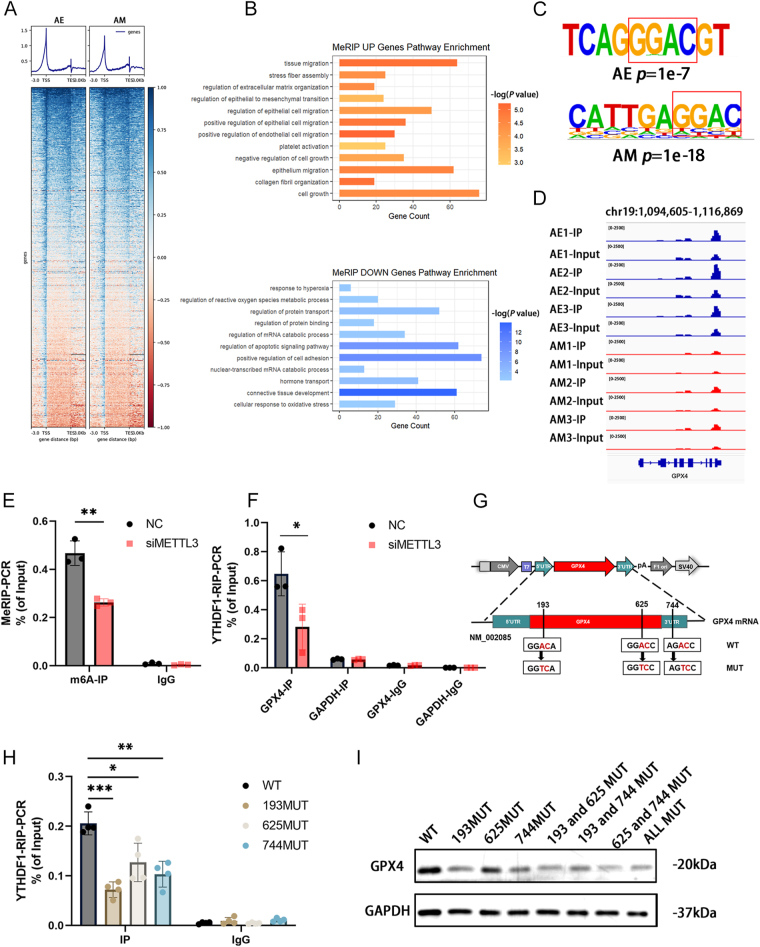
Downregulation of m^6^A-methylated GPX4 mRNA reduced GPX4 mRNA translation in a YTHDF1-dependent manner. (A) Heatmap and peaks of MeRIP-seq after homogenization in RNA ± 3 kb flanking TSSs in endometrium and myometrial lesion in adenomyosis. (B) GO analysis of upregulated and downregulated m^6^A-modified genes based on MeRIP-seq data. (C) The m^6^A motif detected by the HOMER motif discovery tool with MeRIP-seq data. (D) IGV tracks show the distribution of m^6^A peaks of GPX4. (E) MeRIP-qPCR were detected after METTL3 knockdown (*n* = 3). **P < 0.01. (F) YTHDF1-RIP-qPCR were detected after METTL3 knockdown (*n* = 3). *P < 0.05. (G) Schematic representation of wild-type (GPX4-WT) and different sites of m^6^A mutant (GPX4-Mut) constructs. (H) YTHDF1-RIP-qPCR were detected after single sites of GPX4 mutant (*n* = 4). *P < 0.05, **P < 0.01, ***P < 0.001. (I) Protein levels of GPX4 after different sites of GPX4 mutant. The protein levels normalized to GAPDH are shown in Fig. S2F. AE, adenomyosis endometrium; AM, adenomyosis myometrial lesion.

In the aforementioned experiments, we observed that METTL3 could regulate ferroptosis. Notable disparities in the overall m^6^A level and METTL3 expression were detected between eutopic endometrium and the ectopic lesions. So, we utilized these two kinds of specimens in adenomyosis patients to further study its related mechanisms. We mapped the transcriptome-wide m^6^A methylation profiles by conducting m^6^A-RIP sequencing (MeRIP-seq) analysis. There was a decrease in the total m^6^A distribution in the myometrial lesion when compared with the endometrium group. We discovered a common loss of mRNA methylations near the transcription start sites (TSS), with the peak being significantly lower in myometrial lesion (Fig. 5A). GO enrichment analysis unveiled that genes exhibiting increased m^6^A peaks were linked to cell migration and growth, while those with decreased m^6^A modifications were associated with apoptosis and oxidative stress (Fig. 5B). The consensus m^6^A core motif ‘GGAC’ was enriched within the m^6^A peaks, consistent with the ‘DRACH’ motif (*D* = A/G/U; *R* = A/G; and *H* = U/A/C) (Fig. 5C). m^6^A modification on GPX4 mRNA was shown by Integrative Genomics Viewer (IGV) software. As expected, the peaks in the myometrial lesion were much lower (Fig. 5D). Further MeRIP-PCR analysis on endometrial tissue from control and adenomyosis groups confirmed the downregulation of m^6^A-methylated GPX4 (Fig. S2B).

YTH N6-methyladenosine RNA binding protein F1 (YTHDF1), a critical m^6^A reader, facilitates the translation of m^6^A-methylated mRNAs and recruits translation initiation factors to notably impact translation efficiency. Previous experimental results have shown that the mRNA expression level of GPX4 was not difference after METTL3 knockdown, whereas the protein expression level decreased. MeRIP-seq data displayed that genes with high m^6^A enrichment were upregulated, while those with low m^6^A enrichment were downregulated (Fig. S2A). This led us to hypothesize that the decreased m^6^A modification of GPX4 mRNA may reduce its translation by YTHDF1, consequently diminishing GPX4 protein levels. To verify this hypothesis, we conducted MeRIP-PCR and YTHDF1-RIP-PCR. We observed that m^6^A-methylated GPX4 was downregulated after knocking down METTL3 (Fig. 5E), and YTHDF1 effectively bound to GPX4, with significantly higher enrichment in the control group compared to the METTL3 knockdown group (Fig. 5F). These findings were further confirmed in human tissues (Fig. S2C).

Given that m^6^A methylation is usually enriched in the 5′UTR and 3′UTR regions, we specifically selected the NM_002085 transcript to identify and localize m^6^A sites. First, the possible methylation modification sites 193, 625, and 744 were predicted by SRAMP (Fig. S2D). Mutant plasmids were meticulously designed and constructed for individual sites as well as combinations of sites (Fig. 5G). The immunoprecipitated GPX4 with YTHDF1 was obviously decreased after mutation at site 193 (Figs. 5H and S2E). In addition, western blot analysis indicated a reduction in the protein levels of GPX4 upon mutation at sites 193 or 744 compared to the wild type (Fig. 5I).

In conclusion, the downregulation of METTL3 orchestrated the m^6^A modification of *GPX4* mRNA, resulting in reduced *GPX4* mRNA translation in a YTHDF1-dependent manner, ultimately leading to ferroptosis. Notably, site 193 emerged as particularly significant.

### Prompting ferroptosis or inhibiting METTL3 can aggravate adenomyosis *in vivo*

**Figure 6:**
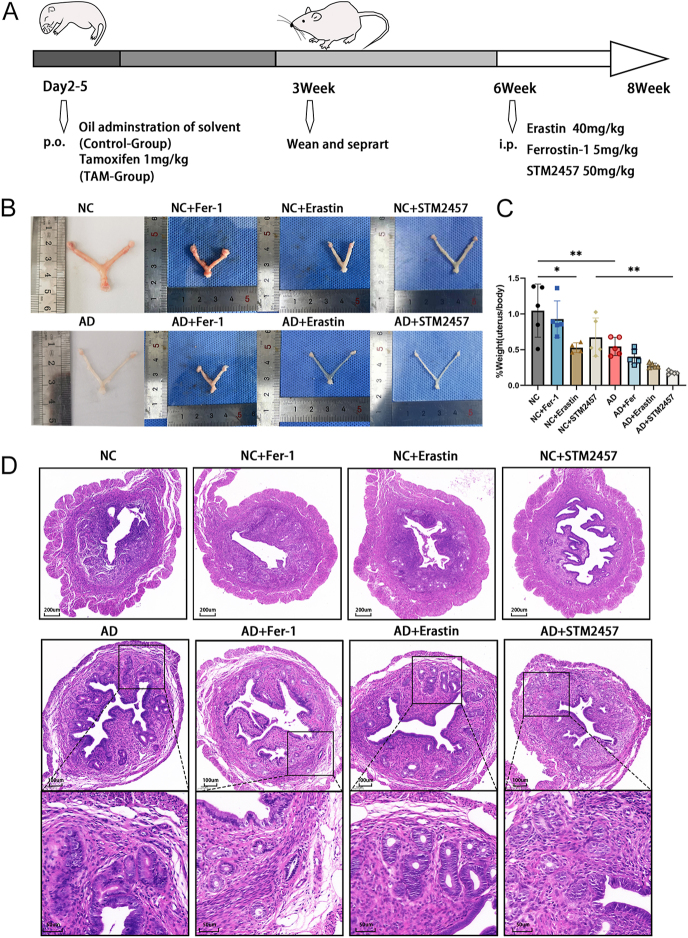
Prompting ferroptosis or inhibiting METTL3 can aggravate adenomyosis *in vivo*. (A) Schematic illustration of the animal experimental design of this study. (B) Uterus weights (in grams) of control mice, adenomyosis mice, and subgroups after treatment with erastin (40 mg/kg), ferrostatin-1 (5 mg/kg), or STM2457 (50 mg/kg) (*n* = 5 in each group except *n* = 4 in the NC + erastin group). *P < 0.05, **P < 0.01, ***P < 0.001. (C) Pictures of the uterus. (D) Cross-section of the uterine horn from each group stained with hematoxylin and eosin. Scale bar = 500 and 200 μm. Squares indicate ectopic endometrial glands. NC, control group; AD, adenomyosis group.

Mouse models of adenomyosis were constructed to determine the role of ferroptosis and METTL3 *in vivo*. Adenomyosis was induced by oral administration of 1 mg/kg tamoxifen from day 2 to day 5 post-birth. The control group and adenomyosis group were each subdivided into three groups for treatment with erastin, ferrostatin-1, or STM2457 (Fig. 6A). Following a 2-week treatment period, a reduction in the uterine weight relative to body weight was observed in the adenomyosis group (Fig. 6B and C). Histological analysis of uterine horns was conducted to confirm the presence of adenomyosis post-tamoxifen administration. In the control group, complete muscle layers were noted in the myometrium, while the adenomyosis group exhibited pronounced uterine developmental disorganization, with dispersed glands throughout the uterine wall (Fig. 6D). Moreover, the ferroptosis inducer erastin and METTL3 inhibitor exacerbated the uterine developmental disorder in the adenomyosis group. Conversely, the ferroptosis inhibitor ferrostatin-1 ameliorated the uterine weight and morphological abnormalities. In addition, in the control group, no ectopic endometrium was found no matter the treatment with erastin, ferrostatin-1 or STM2457, suggesting a causal relationship between ferroptosis, m^6^A, and adenomyosis (Fig. 6D).

**Figure 7:**
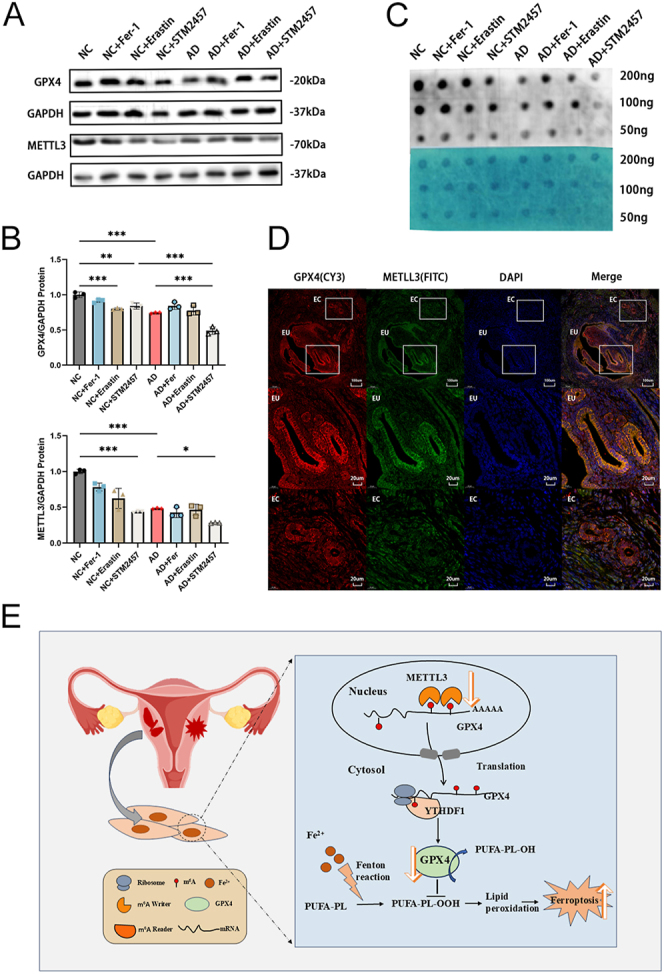
The expression of relevant proteins in the mouse model of adenomyosis. (A) Protein levels of METTL3 and GPX4 from the uterus of eight groups. (B) The protein levels normalized to GAPDH. *P < 0.05, **P < 0.01, ***P < 0.001. (C) Dot blot assay using m^6^A antibody with RNA extracted from the uterus of eight groups. (D) Representative immunofluorescence (IF) staining for METTL3 and GPX4 in a model mouse. Squares indicate eutopic and ectopic endometrial glands. Scale bar = 100 and 20 μm. (E) Schematic representation of the mechanism through the METTL3/YTHDF1/GPX4 axis activating ferroptosis. EU, eutopic endometrium; EC, ectopic lesions.

The western blotting results were consistent with human specimens, indicating a downregulation of METTL3 and GPX4 in adenomyosis. In addition, GPX4 levels were reduced following treatment with STM2457 (Fig. 7A and B). Inhibiting METTL3 led to a decrease in m^6^A methylation levels. Conversely, promoting or inhibiting ferroptosis did not alter the overall level of m^6^A methylation (Fig. 7C). Immunofluorescence imaging demonstrated that the ectopic endometrium in mice exhibited lower expression levels of GPX4 and METTL3 in the adenomyosis group (Fig. 7D).

In summary, this study elucidates the occurrence of ferroptosis in both the endometrium and myometrium in cases of adenomyosis. We provide insight into the mechanism involving the METTL3/YTHDF1/GPX4 axis in the activation of ferroptosis (Fig. 7E). These findings lay the foundation for potential therapeutic approaches targeting adenomyosis.

## Discussion

Ferroptosis, a novel form of programmed cell death, has not been extensively investigated in adenomyosis. In our research, we found that ferroptosis was present in both eutopic and ectopic endometrial tissues of individuals with adenomyosis by detecting changes in mitochondrial morphology, elevated MDA levels, an increased proportion of Fe^2+^ in total iron, and reduced GPX4 protein expression. GPX4 is the most critical anti-lipid peroxidation enzyme in cells. GPX4 can convert toxic polyunsaturated fatty acid phospholipid hydroperoxide into non-toxic polyunsaturated fatty acid phospholipid alcohol, thereby resisting membrane damage and further ferroptosis (Yang *et al.* 2014, Wang *et al.* 2023*b*). A recent study revealed an inverse correlation between ferritin and GPX4 levels in endometriosis (Nulianti *et al.* 2025). But this prior work did not investigate the relationship between GPX4 expression and clinical disease progression. In our research, we found that GPX4 protein expression has negative correlations with CA125 levels, uterine size, and dysmenorrhea severity in adenomyosis. GPX4 may serve as a biomarker for evaluating the severity of adenomyosis.

There was no doubt that m^6^A RNA modification is downregulated in adenomyosis and endometriosis (Zhai *et al.* 2020*a*, Chen *et al.* 2022, Huang & Chen 2023). As for m^6^A regulators, METTL3 is considered the key protein for regulating a variety of different biological processes by targeting different genes, including proliferation and invasion (Li *et al.* 2021*a*, Shen *et al.* 2023), senescence (Wang *et al.* 2023*a*), decidualization of endometrial stromal cells (Li *et al.* 2023), and uterine receptivity (Wan *et al.* 2023). In this study, we provide novel insights into the relationship between m^6^A modification and ferroptosis in adenomyosis. Consistent with previous research, our study confirms that downregulation of METTL3 enhances cell proliferation in EuESCs. However, we have shown that silencing METTL3 can potentiate ferroptosis, supported by elevated levels of MDA and ROS. While these observations appear contradictory, they suggest that METTL3 may play distinct roles in the regulatory pathways of cell proliferation and ferroptosis through m^6^A modifications of different gene sets. Our investigation is solely focused on ferroptosis and its regulatory mechanisms. To gain a more comprehensive understanding, further extensive or in-depth research is warranted to elucidate the impact of METTL3 and ferroptosis on the progression of adenomyosis.

The effects of m^6^A on targeted mRNAs are related to the functions of various mRNA readers. Most readers exert a positive regulatory effect on gene expression. For example, YTHDC1 facilitates RNA processing and export (Roundtree *et al.* 2017). IGF2BP1/2/3 contribute to mRNA stabilization (Huang *et al.* 2018), and YTHDF1 acts as a translation promoter, whereas YTHDF2 lowers mRNA stability (Du *et al.* 2016). Relevant experiments showed that FTO regulated GPX4 in an m^6^A-YTHDF2 dependent manner to suppress colon cancer progression (Zhang *et al.* 2023, Qiao *et al.* 2024). We focused our attention on YTHDF1. As anticipated, we confirmed that YTHDF1 can interact with GPX4, and the enrichment of GPX4 was reduced in adenomyosis. In addition, we successfully identified the correct m^6^A site on GPX4. Through our research, we have elucidated a comprehensive pathway delineating how METTL3 modulates ferroptosis in the context of adenomyosis.

In terms of potential therapies based on ferroptosis or m^6^A methylation, Li reported regression of ectopic lesions following treatment with 40 mg/kg erastin for 2 weeks in a mouse model of endometriosis (Li *et al.* 2021*b*). Wan observed that ectopic lesion volumes were reduced after treatment with 20 mg/kg erastin for 7 days (Liang *et al.* 2022). In our study, we observed worsening disorganization of the uterine structure following treatment with 40 mg/kg erastin in a mouse model of adenomyosis. Ferroptosis inhibitor ferrostatin-1 alleviated the disorder of uterine weight and morphology. Most studies use tamoxifen (Shen *et al.* 2016, Bourdon *et al.* 2021, 2024) or pituitary engraftment (Chen *et al.* 2023) to construct a mouse model of adenomyosis. But unlike enlarged uterus and thick uterine myometrium in humans, this discrepancy arises as myometrial changes precede the invasion of endometrial cells, resulting in a thinner uterine myometrium in the mouse model (Marquardt *et al.* 2020). The possible therapies based on ferroptosis in adenomyosis still have a long way to go because of the lack of a proper animal model and more comprehensive research. In addition, STM2457 was first explored in acute myeloid leukemia (Yankova *et al.* 2021) and has shown therapeutic effects in various malignant tumors (Pan *et al.* 2023, Sun *et al.* 2023). However, in adenomyosis, it aggravated the severity of the condition, as more and larger ectopic lesions were found.

This study has certain limitations. While we collected a substantial number of clinical tissue samples, the limited specimen volume from each case restricted sample sizes for each individual experiment. Notably, endometrial specimens were not stratified by menstrual cycle phase. Consequently, it remains unclear whether proliferative and secretory phase differences influence ferroptosis susceptibility in adenomyotic lesions. Prior studies on apoptosis suggest that ectopic endometrial tissue in adenomyosis and endometriosis loses responsiveness to cyclic hormonal fluctuations during the menstrual cycle (Jones *et al.* 1998). Therefore, whether ferroptosis levels vary across menstrual phases warrants further investigation, particularly given the potential hormonal modulation of cell death pathways.

Overall, our study provides evidence of ferroptosis in both the endometrium and myometrium of individuals with adenomyosis, characterized by reduced RNA m^6^A modification levels and diminished GPX4 protein expression. This research enhances comprehension of the interplay between m^6^A modification and ferroptosis in adenomyosis, offering a basis for developing therapeutic strategies aimed at modulating ferroptosis.

## Conclusion

Ferroptosis has been identified in both eutopic and ectopic lesions of adenomyosis, which is characterized by low GPX4 expression. GPX4 may serve as a biomarker reflecting the severity of adenomyosis due to its significant negative correlation with CA125 levels, uterine size, and the severity of dysmenorrhea in patients. In addition, the overall m^6^A modification level of total RNA is decreased, and reduced expression of METTL3 enhances ferroptosis in endometrial stromal cells. Mechanistically, the downregulation of METTL3 leads to a reduction in m^6^A modification of GPX4 mRNA, resulting in decreased binding of YTHDF1 to GPX4, thereby affecting GPX4 mRNA translation. Promoting ferroptosis or reducing m^6^A methylation levels exacerbates the progression of adenomyosis, while inhibiting ferroptosis may offer therapeutic benefits.

## Supplementary materials



## Declaration of interest

The authors declare that there is no conflict of interest that could be perceived as prejudicing the impartiality of the work reported.

## Funding

This research was supported by the Natural Science Foundation of Henan Provincehttps://doi.org/10.13039/501100006407 (242300421278).

## Author contribution statement

YT performed the majority of the experiments, analyzed the data, interpreted the results, and drafted the manuscript. ZL designed this study, analyzed the data, interpreted the results, and revised the manuscript. XZ, XN, and SN collected specimens. LL, Zh L, JZ, and QZ revised the manuscript. LH designed this study and revised the manuscript. All authors have subsequently reviewed and approved the final version.

## Data availability

The raw sequence data reported in this paper have been deposited in the Genome Sequence Archive (Genomics, Proteomics & Bioinformatics 2021) in the National Genomics Data Center (Nucleic Acids Res 2022), China National Center for Bioinformation/Beijing Institute of Genomics, Chinese Academy of Sciences (GSA-Human: HRA011592), that are publicly accessible at https://ngdc.cncb.ac.cn/gsa-human. The other data underlying this article will be shared on reasonable request to the corresponding author.
