# Supplementary material for: METTL3-mediated m6A modification promotes ferroptosis in adenomyosis through GPX4 in a YTHDF1-dependent manner
Source: Reproduction. 2025 Sep 19;170(4):e250251. doi: 10.1530/REP-25-0251 (PMC12449703; doi:10.1530/REP-25-0251)

Supplementary Table S1

Information of patients

	Control group (n=35)	Adenomyosis group (n=40)
Diagnosis		
AUB	22	/
Uterine leiomyoma	10	/
Cervical intraepithelial neoplasia	3	/
Surgical approach		
Hysteroscopic surgery	22	/
Combined hysteroscopic and laparoscopic surgery	4	8
Laparoscopic Hysterectomy	9	32
Endometrial phase		
Proliferative phase	22	23
Secretory phase	9	9
Pathological result is not shown	4	8

Supplementary Table S2

Baseline characteristics of patients

	**Control group (n=35)**	**Adenomyosis group (n=40)**	**Test statistic (χ2/W)**	***P* value**
Age	45(42~49)	48(44~51)	χ2=5.833	0.054
≤35	3(8.6%)	1(2.5%)		
35 < x≤45	16(45.7%)	10(25.0%)		
45 < x≤55	16(45.7%)	29(72.5%)		
BMI	22.95(20.82~25.71)	24.97(23.14~27.71)	χ2=4.571	0.102
≤18.5	2(5.7%)	0(0.0%)		
18.5 < x≤25	23(65.7%)	21(52.5%)		
>25	10(28.6%)	19(47.5%)		
Gravidity	3(2~4)	4(3~5)	χ2=10.492	0.033*
0	1(2.9%)	0(0.0%)		
1	6(17.1%)	1(2.5%)		
2	6(17.1%)	8(20.0%)		
3	12(34.3%)	8(20.0%)		
> 3	10(28.6%)	23(57.5%)		
Abortions	1(0~2)	2(1~3)	χ2=10.839	0.028*
0	15(42.9%)	6(15.0%)		
1	9(25.7%)	8(20.0%)		
2	8(22.9%)	14(35.0%)		
3	1(2.9%)	6(15.0%)		
> 3	2(5.7%)	6(15.0%)		
Anemia (Hb < 110g/L)	15(42.9%)	26(65.0%)	χ2=3.693	0.055
Dysmenorrhea	0(0~2)	3(0~4)	χ2=17.594	< 0.001*
VAS=0	25(71.4%)	11(27.5%)		
1≤VAS≤3	9(25.7%)	16(40.0%)		
4≤VAS≤6	1(2.9%)	8(20.0%)		
7≤VAS≤10	0(0.0%)	5(12.5%)		

CA125	12.50(9.62~17.10)	59.20(38.75~110.74)	χ2=39.039	< 0.001*
≤35U/mL	33(94.3%)	9(22.5%)		
>35U/mL	2(5.7%)	31(77.5%)		
CA199	10.10(4.97~13.30)	9.34 (5.96~15.42)	χ2=0.797	0.372
≤37U/mL	34(97.1%)	37(92.5%)		
>37U/mL	1(2.9%)	3(7.5%)		
Uterine size (L*W*A cm^3)	163.99(93.86~247.08)	379.55(272.18~611.20)	W=141.000	< 0.001*

Notations: L: length diameter; W: width diameter; A: Anteroposterior diameter, Mann–Whitney U test (represented by W), chi-square test (represented by χ2),**P* < 0.05

Supplementary Table S3

The sequences of si-RNA

	**sense strand (5'-3')**	**antisense strand (5'-3')**
Si-METTL3	GCACUUGGAUCUACGGAAUTT	AUUCCGUAGAUCCAAGUGCTT
Si-NC	UUCUCCGAACGUGUCACGUTT	ACGUGACACGUUCGGAGAATT

Supplementary Table S4

Primer sequences for RT-qPCR and MeRIP-PCR

	**Forward(5'-3')**	**Reverse(5'-3')**
GAPDH	GGAGCGAGATCCCTCCAAAAT	GGCTGTTGTCATACTTCTCATGG
METTL3	TTGTCTCCAACCTTCCGTAGT	CCAGATCAGAGAGGTGGTGTAG
GPX4 for RT-qPCR	TTCCCGTGTAACCAGTTCGG	GTGGAGAGACGGTGTCCAAA
GPX4 for MeRIP-PCR	CTCCCAGTGAGGCAAGACCGAA	TATCGAATTTGACGTTGTAGCCCG

Supplementary figure S1

(A) Prussian blue staining of the control endometrium, adenomyosis endometrium and myometrial lesion of adenomyosis. Scale bar=100μm. (B) Immunofluorescent staining of EuESCs using vimentin (red) and cytokeratin 1(green). Scale bar=200um. (C) The protein level normalized to GAPDH. * *P*< 0.05, ***P*< 0.01. Notations: CE: Control Endometrium; AE: Adenomyosis Endometrium; AM: Adenomyosis Myometrial Lesion.

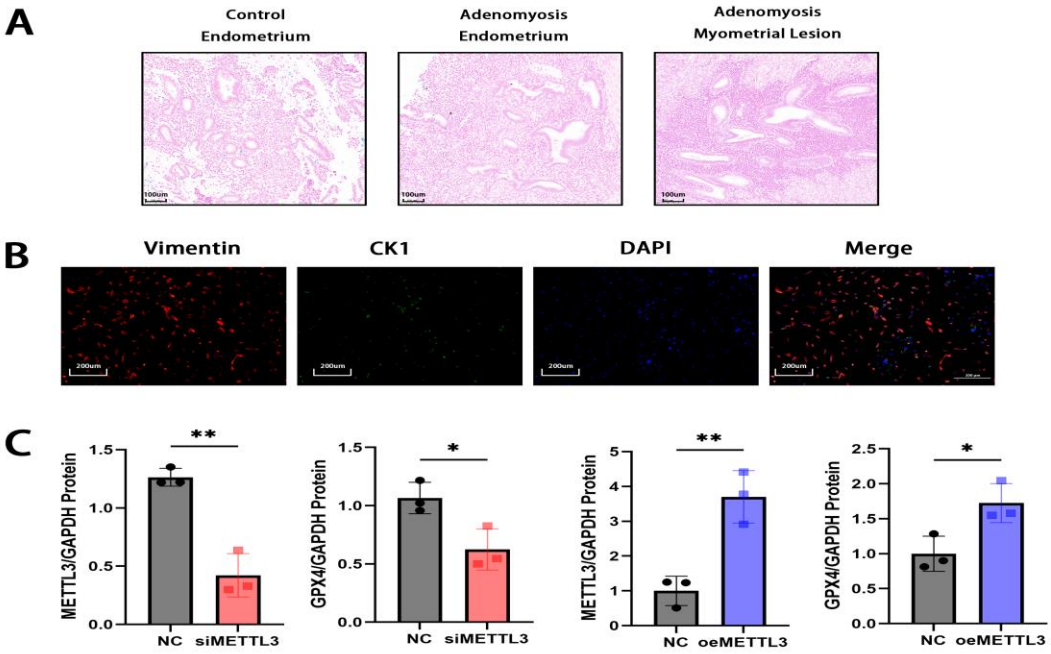

Supplementary figure S2

(A) Genes expression with different m^{6}A enrichment by MeRIP-seq. * *P*< 0.05 (B) MeRIP-qPCR of endometrium tissue of control groups and adenomyosis patients. (n=3) **P*< 0.05. (C) YTHDF1-RIP-qPCR of endometrium tissue of control groups and adenomyosis patients. (n=4). ***P* < 0.01. (D) The m^{6}A methylation sites of GPX4 were obtained from SRAMP database. (E) YTHDF1-RIP-qPCR were detected after mixed sites of GPX4 mutant (n=4). ****P*< 0.001. (F) The protein level normalized to GAPDH. * *P*< 0.05, ****P*< 0.001.

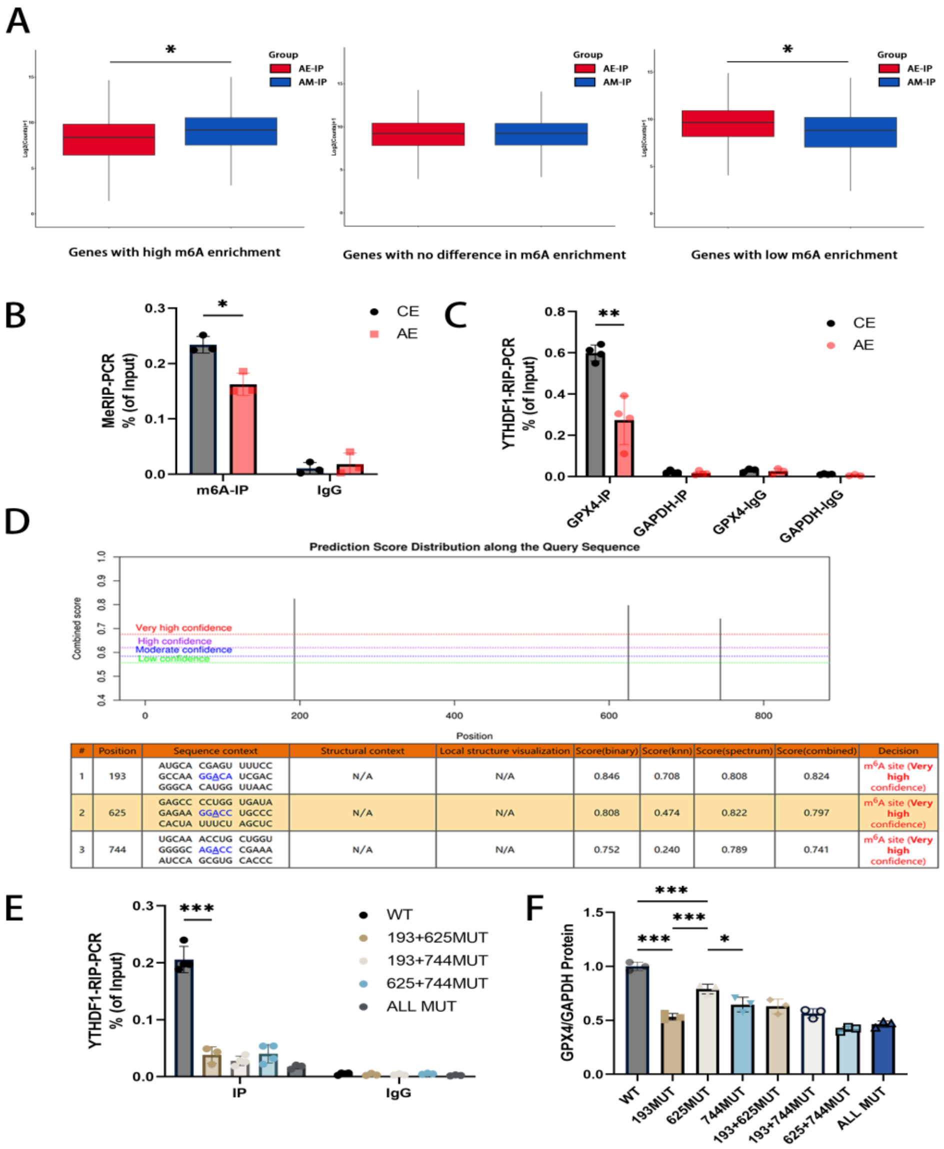

Supplementary figure S3

The uncropped western blot images are shown. Each treatment group included at least three replicates and each experiment was performed with three repetitions. The results marked with rectangles are those presented in the original text.

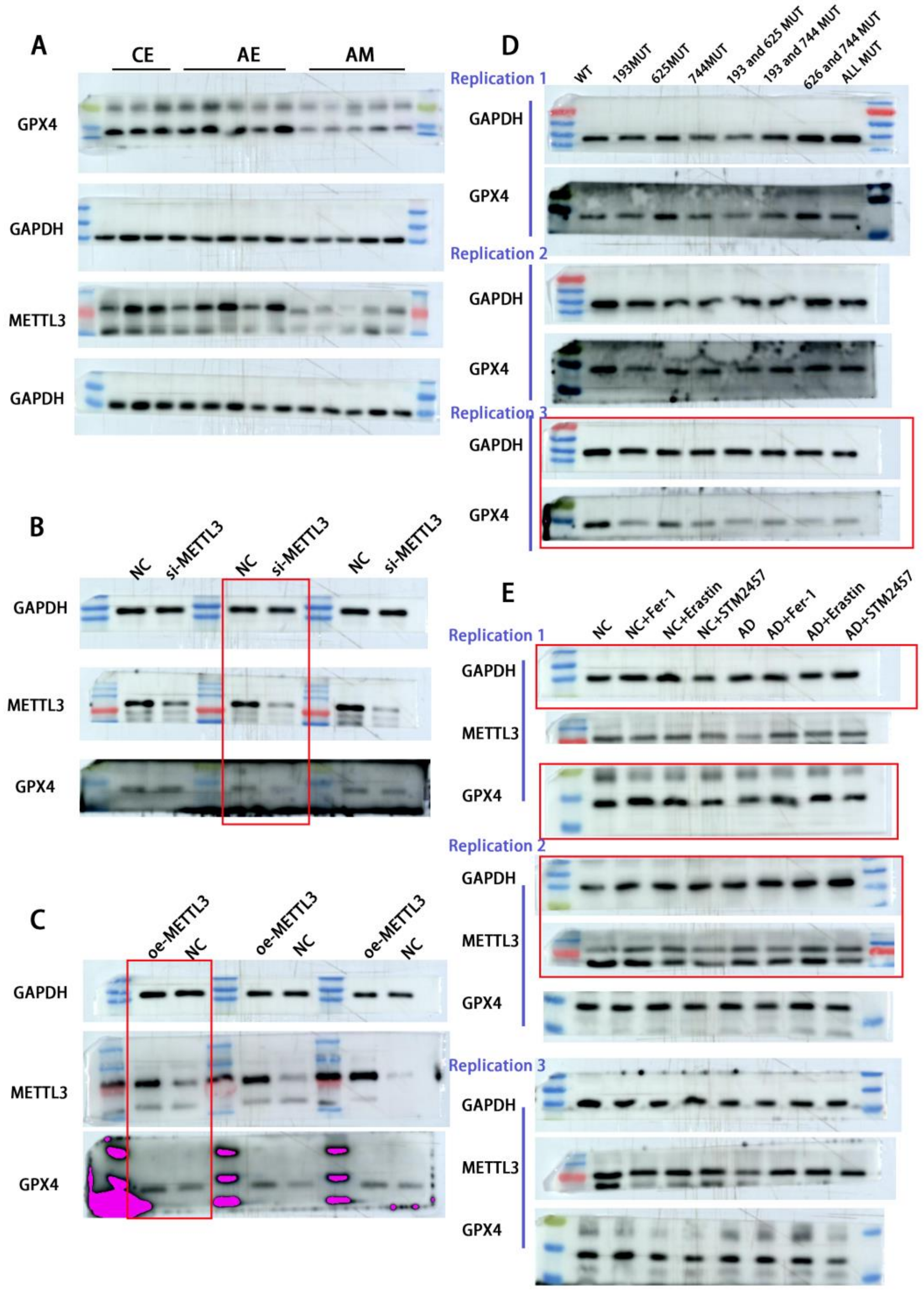

Supplement: Supplementary file 1 [file supplementary_materials.pdf]
